# Morphological and phylogenetic analyses reveal two new entomopathogenic fungi within the genus *Ophiocordyceps* (*Ophiocordycipitaceae*, *Hypocreales*)

**DOI:** 10.3897/mycokeys.129.177387

**Published:** 2026-03-12

**Authors:** Zhi-Qin Wang, Zi-Han Wang, Wen-Ya Shao, Yuan-Bing Wang

**Affiliations:** 1 College of Biological Science and Food Engineering, Southwest Forestry University, Kunming, 650224, China State Key Laboratory of Phytochemistry and Natural Medicine, Kunming Institute of Botany, Chinese Academy of Sciences Kunming China; 2 State Key Laboratory of Phytochemistry and Natural Medicine, Kunming Institute of Botany, Chinese Academy of Sciences, Kunming 650201, China Yunnan Key Laboratory for Fungal Diversity and Green Development, Kunming Institute of Botany, Chinese Academy of Sciences Kunming China; 3 Yunnan Key Laboratory for Fungal Diversity and Green Development, Kunming Institute of Botany, Chinese Academy of Sciences, Kunming 650201, China College of Biological Science and Food Engineering, Southwest Forestry University Kunming China

**Keywords:** Morphology, multi-gene phylogeny, new taxa, *
Ophiocordyceps
*

## Abstract

This study reports on two new species, *Ophiocordyceps simaoensis* and *O. guishanensis*, from Yunnan Province, Southwestern China. These two species are introduced based on the morphological comparisons and phylogenetic evidence of a multigene dataset (nr*SSU*, nr*LSU*, *tef-1α*, *rpb1*, and *rpb2*). Phylogenetic analyses reveal that *O. simaoensis* is closely related to *O. longistipes* and *O. fusiformis*, whereas *O. guishanensis* shares a close relationship with *O. acicularis* and *O. liaoningensis*. Morphologically, *O. simaoensis* can be distinguished from its phylogenetically close relatives by its smaller, immersed perithecia, shorter asci and ascospores. *Ophiocordyceps guishanensis* differs from *O. acicularis* and *O. liaoningensis* by its smaller asci.

## Introduction

Based on morphological and multi-gene phylogenetic analyses of 162 taxa, Sung et al. (2007) reclassified *Cordyceps* Fr. *s. l*. into three families and four genera. They were *Cordyceps* s.s. within *Cordycipitaceae*, *Ophiocordyceps* Petch and *Elaphocordyceps* G.H. Sung & Spatafora within *Ophiocordycipitaceae*, and *Metacordyceps* G.H. Sung et al. within *Clavicipitaceae* s.s. (Sung et al. 2007). Since this taxonomic revision, *Ophiocordyceps* has been resumed and recognized as the type genus of *Ophiocordycipitaceae*, forming a sister clade with the genus *Elaphocordyceps* (Sung et al. 2007). *Ophiocordyceps* was originally established by Petch based on the type species, *O. blattae* Petch, and included three species: *O. unilateralis* (Tul. & C. Tul.) Petch, *O. peltata* (Wakef.) Petch, and *O. rhizoidea* (Höhn.) Petch. It was to accommodate species with thickened ascus caps and ascospores that lack septa and do not break into secondary ascospores at maturity (Petch 1931).

*Ophiocordyceps* species are distributed globally, with tropical and subtropical regions currently harboring the highest number of documented species (Lao et al. 2021). This genus represents the largest one within the family *Ophiocordycipitaceae*, with approximately 400 records in the Index Fungorum (http://www.indexfungorum.org/, Jan. 27, 2026) (Araújo et al. 2018; Luangsa-ard et al. 2018). Members of this genus are morphologically diverse, with the position, shape, and color of the fertile structures serving as the key diagnostic features (Mongkolsamrit et al. 2019). Stromata are typically dark-colored, fibrous, and range from hard to flexible in texture; perithecia are superficial to immersed and are arranged vertically or obliquely (Sung et al. 2007). Ascospores are usually cylindrical, septate, and break into secondary ascospores or remain intact after maturation (Ban et al. 2015; Sanjuan et al. 2015; Luangsa-ard et al. 2018). Anamorphs associated with the genus include *Hirsutella* Pat., *Hymenostilbe* Petch, *Sorosporella* Sorokin, *Stilbella* Lindau, *Syngliocladium* Petch (Quandt et al. 2014), among which *Hirsutella* and *Hymenostilbe* are the most common and representative taxa (Maharachchikumbura et al. 2016).

In the previous phylogenetic study, Sung et al. (2007) divided *Ophiocordyceps* into two major clades: the *C. sphecocephala* (Klotzsch ex Berk.) Berk. & M.A. Curtis clade and *C. unilateralis* (Tul. & C. Tul.) Sacc. clade. Based on a five-gene (nr*SSU*, nr*LSU*, *tef-1α*, *rpb1*, and *rpb2*) phylogeny from 121 taxa, Sanjuan et al. (2015) divided *Ophiocordyceps* into four clades, namely the *O. ravenelii*, *O. unilateralis*, *O. sobolifera*, and *O. sphecocephala* clades (Sanjuan et al. 2015). Subsequently, Wang et al. (2018) refined the phylogeny of *Ophiocordyceps* based on 96 taxa, dividing it into four clades: the *Hirsutella*, *O. sobolifera*, *O. ravenelii*, and *O. sphecocephala* clades. They further subdivided the *Hirsutella* clade into six subclades: *H. sinensis*, *H. citriformis*, *H. guyana*, *H. nodulosa*, *H. thompsonii*, and *Hirsutella* ant pathogen subclade (Wang et al. 2018). More recently, Xu et al. (2025) analyzed 213 taxa and designated four clades within *Ophiocordyceps*: the *Hirsutella*-like A, *Hirsutella*-like B, *O. nutans*, and *O. ravenelii* clades. These studies highlight that the infrageneric classification of *Ophiocordyceps* continues to change as taxon sampling and molecular datasets expand.

During investigations of entomopathogenic fungal diversity in the Yunnan Province of China, specimen collections were conducted, and fungal strains were isolated and purified. Through a combination of multi-gene phylogenetic analyses and morphological examination, two new species of *Ophiocordyceps* are introduced: *O. simaoensis* and *O. guishanensis*. This study provides detailed morphological descriptions, illustrations, and comparisons with closely related taxa for both new species.

## Materials and methods

### Fungal collection and isolation

Specimens were collected from Shilin County (Kunming City), and Simao District (Pu’er City), Yunnan Province, China. After collection, specimens were placed in sterilized plastic tubes, stored at 4 °C, and transported to the laboratory. The detailed procedure for obtaining axenic cultures of samples followed the description of Wang et al. (2020). They were cultured on PDA plates containing 0.1 g/L chloramphenicol. Specimens were deposited in the Cryptogamic Herbarium of the Kunming Institute of Botany, Chinese Academy of Sciences (**KUN-HKAS**). Cultures were deposited in the Kunming Institute of Botany Culture Collection (**KUNCC**), Chinese Academy of Sciences.

### Morphological observations

Specimens were examined in the laboratory using the Olympus SZ60 stereo microscope. Macro-morphological characteristics, including stromata size, texture, shape, and colour, were measured and recorded. Stromata were sectioned, and the sections were placed on a slide, either dripping with water or stained with lactic acid-cotton blue, for microscopic observations under an Olympus BX53 microscope. Sizes and shapes of perithecia, asci, and ascospores were documented. In order to observe and record the characteristics of pure cultures (texture and colour), several subcultures were transferred from purified colonies onto fresh PDA plates and incubated at 25 °C.

### DNA extraction, PCR, and sequencing

Genomic DNA was extracted from fresh mycelia grown on PDA or from dried specimens. Before extraction, the specimens were rinsed in 75% alcohol for approximately 10 seconds, immersed in sterile water for 1 minute, and subsequently dried with sterile filter paper. DNA extraction was performed using the Trelief Hi-Pure Plant Genomic DNA Kit (Tsingke Biotechnology Co., Ltd., Beijing, China) according to the manufacturer’s protocol. Five genes (nr*SSU*, nr*LSU*, *tef-1α*, *rpb1*, and *rpb2*) were amplified using the primer pairs referred to in Wang et al. 2020. Amplifications of the nuclear ribosomal small subunits (nr*SSU*), the nuclear ribosomal large subunits (nr*LSU*), the translation elongation factor 1α (*tef-1α*), and the largest and second largest subunits of RNA polymerase II (*rpb1* and *rpb2*) were conducted as described by Wang et al. (2020). Each 25 µL PCR reaction consisted of 12.5 µL of 2 × Taq PCR Master Mix (Tiangen Biotech Co., Ltd., Beijing, China), 8.5 µL of sterile water, 1 µL of each forward and reverse primer and 2 µL of DNA template. The Veriti 96-Well thermal cycler (Life Technologies Holdings Pte Ltd Block, Marsiling Industrial Estate Road 3, Singapore) was used to perform amplification reactions. PCR products were sequenced by the Tsingke Biotechnology Co., Ltd (Kunming, China).

### Sequence alignment and phylogenetic analyses

The newly generated data in this study were checked using MEGA v.6.06 (Tamura et al. 2013). Five genes (nr*SSU*, nr*LSU*, *tef-1α*, *rpb1*, and *rpb2*) were retrieved from GenBank, based on the BLAST results in NCBI and previous studies by Fan et al. (2024) and Xu et al. (2025). The sequence information used for phylogenetic tree construction is listed in Table 1. Each gene dataset was aligned, and poorly aligned regions were excluded using MEGA v.6.06 (Tamura et al. 2013). The concatenation of nr*SSU*, nr*LSU*, *tef-1α*, *rpb1* and *rpb2* genes was performed by Phylosuite v1.2.2 (Zhang et al. 2020). Phylogenetic analyses were conducted using bayesian inference (BI) and maximum likelihood (ML) methods (Ronquist and Huelsenbeck 2003; Stamatakis et al. 2008). The BI and ML trees were constructed using MrBayes v.3.2.2 and IQ-tree v.2.1.3, respectively (Ronquist et al. 2012; Nguyen et al. 2015). The best-fitting likelihood model was selected by Modelfinder in the PhyloSuite v 1.2.2 (Kalyaanamoorthy et al. 2017; Zhang et al. 2020). The optimal models for ML and BI analyses were determined as TIM2+F+I+G4 and GTR+F+I+G4, respectively. Resulting phylogenetic trees were edited using Figtree v.1.4.3 and visualized in Adobe Illustrator CS6.

**Table 1.** Names, voucher information, host and GenBank accession numbers of taxa used in this study.

Species	Voucher	Host	GenBank Accession Number					References
			nr*SSU*	nr*LSU*	*tef1–a*	*rpb1*	*rpb2*	
*Drechmeria balanoides*	CBS 250.82	* Lepidoptera *	AF339588	AF339539	DQ522342	–	DQ522442	Sung et al. 2007
*D. gunnii*	OSC 76404	* Lepidoptera *	AF339572	AF339522	AY489616	AY489650	DQ522426	Kepler et al. 2012
*D. sinensis*	CBS 567.95	–	AF339594	AF339545	DQ522343	DQ522389	DQ522443	Sung et al. 2001
*Hirsutella fusiformis*	ARSEF 5474	* Coleoptera *	KM652067	KM652110	KM651993	KM652033	–	Simmons et al. 2015
*H. gigantea*	ARSEF 30	* Hymenoptera *	–	JX566977	JX566980	KM652034	–	Simmons et al. 2015
*H. guyana*	ARSEF 878	*Hemiptera*: *Cicadellidae*	KM652068	KM652111	KM651994	KM652035	–	Simmons et al. 2015
*H. illustris*	ARSEF 5539	* Hemiptera *	KM652069	KM652112	KM651996	KM652037	–	Simmons et al. 2015
*H. kirchneri*	ARSEF 5551	*Acari*: *Eriophyidae*	KM652070	KM652113	KM651997	–	–	Simmons et al. 2015
*H. kuankuoshuiensis*	GZUIFR-2012KKS3-1	Larva of *Lepidoptera*	–	KY415582	KY415590	KY945360	–	Qu et al. 2021
*H. lecaniicola*	ARSEF 8888	*Hemiptera*: *Coccidae*	KM652071	KM652114	KM651998	KM652038	–	Simmons et al. 2015
*H. liboensis*	ARSEF 9603	*Lepidoptera*: *Cossidae*	KM652072	KM652115	–	–		Simmons et al. 2015
*H. nodulosa*	ARSEF 5473	*Lepidoptera*: *Pyralidae*	KM652074	KM652117	KM652000	KM652040	–	Simmons et al. 2015
*H. radiata*	ARSEF 1369	* Diptera *	KM652076	KM652119	KM652002	KM652042	–	Simmons et al. 2015
*H. repens* nom. inval.	ARSEF 2348	*Hemiptera*: *Delphacidae*	KM652077	KM652120	KM652003	–	–	Simmons et al. 2015
*H. rhossiliensis*	ARSEF 3751	–	KM652081	KM652124	KM652007	KM652046	–	Simmons et al. 2015
*H. rhossiliensis*	ARSEF 2931	*Tylenchida*: *Delphacidae*	KM652078	KM652121	KM652004	KM652043	–	Simmons et al. 2015
*H. rhossiliensis*	ARSEF 3207	–	KM652079	KM652122	KM652005	KM652044	–	Simmons et al. 2015
*H. rhossiliensis*	ARSEF 3747	*Tylenchida*: *Criconematidae*	KM652080	KM652123	KM652006	KM652045	–	Simmons et al. 2015
*H. satumaensis*	ARSEF 996	*Lepidoptera*: *Pyralidae*	KM652082	KM652125	KM652008	KM652047	–	Simmons et al. 2015
*H. sublata*	ARSEF 2227	* Lepidoptera *	KM652086	KM652130	KM652013	KM652051	–	Simmons et al. 2015
*H. thompsonii*	ARSEF 256	–	KM652090	KM652135	KM652018	KM652053	–	Simmons et al. 2015
*H. versicolor*	ARSEF 1037	*Hemiptera*: *Membracidae*	KM652102	KM652150	KM652029	KM652063	–	Simmons et al. 2015
*H. vnecatrix*	ARSEF 5549	* Ixodida *	KM652073	KM652116	KM651999	KM652039	–	Simmons et al. 2015
*H. cryptosclerotium*	ARSEF 4517	* Hemiptera *	KM652066	KM652109	KM651992	KM652032	–	Simmons et al. 2015
*H. strigose*	ARSEF 2197	*Hemiptera*: *Cicadellidae*	KM652085	KM652129	KM652012	KM652050	–	Simmons et al. 2015
*Ophiocordyceps acicularis*	OSC 110987	* Coleoptera *	EF468950	EF468805	EF468744	EF468852	–	Sung et al. 2007
*O. acicularis*	OSC 110988	* Coleoptera *	EF468951	EF468804	EF468745	EF468853	–	Sung et al. 2007
*O. agriota*	ARSEF 5692	* Coleoptera *	DQ522540	DQ518754	DQ522322	DQ522368	DQ522418	Kepler et al. 2012
*O. alboperitheciata*	YHH 16775	* Lepidoptera *	–	MT222278	MT222279	MT222280	MT222281	Fan et al. 2021
*O. anocheti*	HKAS 132269	Adult of *Anochetus*	PV139233	PV139249	PV156012	PV155983	PV155997	Xie et al. 2025
*O. anocheti*	HKAS 132231	Adult of *Anochetus*	PV139232	PV139248	PV156011	PV155982	PV155996	Xie et al. 2025
*O. anshunensis*	GMBC 3027	Adult of *Carbula*	–	PP577939	PP681122	PV681112	PP681117	Guan et al. 2025
*O. anshunensis*	GMBC 3026	Adult of *Carbula*	–	PP577938	PP681121	PV681111	PP681116	Guan et al. 2025
*O. aphodii*	ARSEF 5498	* Coleoptera *	DQ522541	DQ518755	DQ522323	–	DQ522419	Spatafora et al. 2007
*O. appendiculata*	NBRC 106959	* Coleoptera *	JN941729	JN941412	AB968578	JN992463	AB968540	Ban et al. 2015
*O. appendiculata*	NBRC 106960	* Coleoptera *	JN941728	JN941413	AB968577	JN992462	AB968539	Ban et al. 2015
*O. araracuarensis*	HUA 186148	–	KC610790	KF658679	KC610739	KF658667	KC610717	Dai et al. 2024
*O. arborescens*	NBRC 105890	*Lepidoptera*: *Cossidae*	–	AB968415	AB968573	–	AB968535	Ban et al. 2025
*O. arborescens*	NBRC 105891	*Lepidoptera*: *Cossidae*	–	AB968414	AB968572	–	AB968534	Ban et al. 2015
*O. bannaensis*	GACP24-0801	* Camponotus *	–	–	PQ880174	PV880176	PP880175	Hongsanan et al. 2025
*O. asiatica*	BCC 30516	Termite	–	MH753675	MK284263	MK214105	MK214091	Tasanathai et al. 2019
*O. asiatica*	BCC 86435	Termite	–	MH753676	–	MK214105	MK214092	Tasanathai et al. 2019
*O. blackebarnesii*	MISSOU1	–	KX713644	–	KX713686	KX713713	–	Araújo et al. 2018
*O. blackebarnesii*	MISSOU3	–	KX713643	KX713608	KX713687	KX713714	–	Araújo et al. 2018
*O. brunneanigra*	BCC 69032	–	–	MF614654	MF614638	MF614668	MF614681	Luangsa-ard et al. 2018
*O. brunneirubra*	BCC 14384	Termite	–	MH753690	GU797121	MK751465	MK751468	Tasanathai et al. 2019
*O. brunneaperitheciata*	BCC 64201	–	–	MF614658	MF614643	–	MF614685	Luangsa-ard et al. 2018
*O. brunneaperitheciata*	BCC 49312	* Lepidoptera *	–	MF614660	MF614642	–	MF614686	Luangsa-ard et al. 2018
*O. brunneipunctata*	OCS 128576	*Coleoptera*: *Elateridae*	DQ522542	DQ518756	DQ522324	DQ522369	DQ522420	Spatafora et al. 2007
*O. buquetii*	HMAS 199617	–	KJ878940	KJ878905	KJ878985	KJ879020	–	Quandt et al. 2014
*O. camponoti-balzani*	G104	*Camponotus balzani*	KX713660	KX713593	KX713689	KX713703	–	Araújo et al. 2018
*O. camponoti bispinosi*	OBIS4	–	KX713637	–	KX713692	KX713720	–	Araújo et al. 2018
*O. camponoti-femorati*	FEMO2	–	KX713663	KX713590	KX713678	KX713702	–	Araújo et al. 2018
*O. camponoti-hippocrepidis*	HIPPOC	* Hemiptera *	KX713655	KX713597	KX713673	KX713707	–	Araújo et al. 2018
*O. camponoti-nidulantis*	NIDUL2	–	KX713640	KX713611	KX713669	KX713717	–	Araújo et al. 2018
*O. camponoti-rufipedis*	G108	–	KX713659	KX713594	KX713679	KX713704	–	Araújo et al. 2018
*O. camponoti-renggeri*	ORENG	–	KX713634	KX713617	KX713671	–	–	Araújo et al. 2018
*O. cf. acicularis*	OSC 128580	* Coleoptera *	DQ522543	DQ518757	DQ522326	DQ522371	DQ522432	Spatafora et al. 2007
*O. clavata*	NBRC 106961	–	–	JN941414	AB968586	–	AB968547	Schoch et al. 2012
*O. coccidiicola*	NBRC 100682	–	AB968391	AB968419	AB968583	–	AB968545	Ban et al. 2015
*O. cochlidiicola*	HMAS 199612	–	KJ878917	KJ878884	KJ878965	KJ878998	–	Quandt et al. 2014
*O. communis*	BCC 1842	Termite	–	MH753680	MK284266	MK214110	MK214096	Tasanathai et al. 2019
*O. communis*	BCC 1874	Termite	–	MH753679	MK284267	MK214109	MK214095	Tasanathai et al. 2019
*O. communis*	BCC 2754	Termite	–	MH753681	MK284268	MK214111	MK214097	Tasanathai et al. 2019
*O. crinalis*	GDGM 17327	* Lepidoptera *	KF226253	KF226254	KF226256	KF226255		Wang et al. 2014
*O. curculionum*	OSC 151910	–	KJ878918	KJ878885	–	KJ878999	–	Quandt et al. 2014
*O. cystidiata*	GZUIFR-2023XY-OA5	* Hepialidae *	PQ497594	PQ497634	–	PQ516632	PQ516636	Xu et al. 2025
*O. cystidiata*	GZUIFR-2023XY- OA5C	* Hepialidae *	PQ497595	PQ497635	–	PQ516633	PQ516637	Xu et al. 2025
*O. desmidiospora*	SJS3Des	–	MH536515	MH536514	MN785129	MN785131	–	Saltamachia and Araújo 2020
*O. duyunensis*	HKAS 125843	Larva of *Lepidoptera*	–	OQ110570	OQ116920	OQ116923	–	Manawasinghe et al. 2025
*O. duyunensis*	HKAS 125849	Cocoon of *Lepidoptera*	–	OQ110571	OQ116921	OQ116924	–	Manawasinghe et al. 2025
*O. duyunensis*	HKAS 125850	Cocoon of *Lepidoptera*	–	OQ110572	OQ116922	OQ116925	–	Manawasinghe et al. 2025
*O. elongata*	OCS 110989	* Lepidoptera *	–	EF468808	EF468748	EF468856	–	Sung et al. 2007
*O. entomorrhiza*	KEW 53484	–	EF468954	EF468809	EF468749	EF468857	EF468911	Sung et al. 2007
*O. evansii*	Ophsp 858	* Lepidoptera *	KC610796	KC610770	KC610736	KP212916	–	Sanjuan et al. 2015
*O. fenggangensis*	FG21042850	* Lepidoptera *	OR527538	OR527541	OR526345	OR526350	OR526353	Peng et al. 2024
*O. fenggangensis*	HKAS 125848	* Lepidoptera *	–	OR527542	OR526346	OR526351	–	Peng et al. 2024
*O. floriformis*	BBH 51295	* Clephydroneura *	–	PV257643	PV274276	–	PV274287	Mongkolsamrit et al. 2025
*O. formicarum*	TNS F18565	–	KJ878921	KJ878888	KJ878968	KJ879002	KJ878946	Quandt et al. 2014
*O. formosana*	MFLU:15-3888	–	KU854951	–	KU854949	KU854947	–	Li et al. 2016
*O. formosana*	NTU 00035	–	–	–	KT275192	KT275190	KT275191	Wang et al. 2015a
*O. forquignonii*	OSC 151902	–	KJ878912	KJ878876	–	KJ878991	KJ878945	Quandt et al. 2014
*O. fuigoromorphila*	Ophara 729	–	KC610795	KC610761	KC610730	KF658677	–	Sanjuan et al. 2015
*O. fusiformis*	BCC 93025	Termite	–	MZ675422	MZ707849	MZ707855	MZ707805	Tasanathai et al. 2022
*O. geometridicola*	BCC 35947	–	–	MF614647	MF614631	MF614664	MF614678	Luangsa-ard et al. 2018
*O. geometridicola*	BCC 79823	–	–	MF614648	MF614632	MF614663	MF614679	Luangsa-ard et al. 2018
*O. ghanensis*	Gh41	–	KX713656	–	KX713668	KX713706	–	Araújo et al. 2018
*O. globiperitheciata*	HKAS 126130	Termite	OR082950	OR015968	OR030532	OR119834	–	Fan et al. 2024
*O. globiperitheciata*	HKAS 126131	Termite	OR082951	OR015969	OR030533	OR119835	–	Fan et al. 2024
*O. globosa*	BCC 93023	Termite	–	MZ675419	MZ707846	MZ707861	–	Tasanathai et al. 2022
** guishanensis **	**HKAS 126111**	**Larva of *Elateridae (Coleoptera)***	**PX852564**	**–**	**PX893078**	**PX893073**	**–**	**This study**
***O. guishanensis***	**HKAS 126112**	**Larva of *Elateridae (Coleoptera)***	**–**	**PX852567**	**PX893079**	**PX893074**	**PX893071**	**This study**
*O. highlandensis*	HKAS 83207	* Scarabaeoidea *	KM581284	–	–	KM581277	KM581281	Yang et al. 2015
*O. highlandensis*	YHH OH1301	* Melolonthidae *	KR479869	–	KR479870	KR479872	KR479874	Wang et al. 2015b
*O. irangiensis*	OSC 128578	*Hymenoptera*: ant	DQ522556	DQ518770	DQ522345	DQ522391	DQ522445	Spatafora et al. 2007
*O. isopterae*	MY12376	Termite	–	MZ675420	MZ707847	MZ707859	MZ707803	Tasanathai et al. 2022
*O. isopterae*	BCC 93042	Termite	–	MZ675421	MZ707848	–	MZ707804	Tasanathai et al. 2022
*O. jilinensis*	JL2420	* Orthoptera *	PQ528941	PQ528944	PQ522479	–	PQ569912	Cao et al. 2025
*O. jilinensis*	JL2421	* Orthoptera *	PQ528940	PQ528943	PQ522478	PQ522476	PQ569911	Cao et al. 2025
*O. karstii*	MFLU:15-3884	* Hepialidae *	KU854952	–	KU854945	KU854943	–	Li et al. 2016
*O. karstii*	MFLU:15-3885	* Hepialidae *	KU854953	–	KU854946	KU854944	–	Li et al. 2016
*O. keqinii*	HKAS 135614	* Lepidoptera *	PP951447	PP958849	PP956623	PP966946	–	Xie et al. 2025
*O. khokpasiensis*	BCC 48071	Termite	–	MH753682	MK284269	MK214112	–	Tasanathai et al. 2019
*O. khokpasiensis*	BCC 48072	Termite	–	MH753683	MK284270	MK214113	–	Tasanathai et al. 2019
*O. khokpasiensis*	BCC 1764	Termite	–	MH753684	MK284271	MK214114	MK214098	Tasanathai et al. 2019
*O. khonkaenensis*	BCC81463	–	MK632127	MK632102	MK632076	MK632169	MK632158	Crous et al. 2019
*O. kimflemingiae*	SC09B	–	KX713631	–	KX713698	KX713724	–	Araújo et al. 2018
*O. knipofioides*	MF90	* Hymenoptera *	MK874746	MK875538	–	MK863827	–	Araújo et al. 2018
*O. konnoana*	EFCC 7315	* Coleoptera *	EF468959	–	EF468753	EF468861	EF468916	Sung et al. 2007
*O. lanpingensis*	YHOL0707	* Hepialidae *	KC417459	KC417461	KC417463	KC417465	–	Chen et al. 2013
*O. lianggii*	HKAS 125845	* Lepidoptera *	OR527539	OR527543	OR526347	–	–	Peng et al. 2024
*O. lianggii*	LB22071253	* Lepidoptera *	OR527540	OR527544	OR526348	–	OR526354	Peng et al. 2024
*O. liangshanensis*	YFCC 85777	*Lepidoptera*: *Hepialidae*	MT774218	MT774225	MT774246	MT774232	MT774239	Wang et al. 2021
*O. liangshanensis*	YFCC 8578	*Lepidoptera*: *Hepialidae*	MT774219	MT774226	MT774247	MT774233	MT774240	Wang et al. 2021
*O. liaoningensis*	HKAS 132185	Larva of *Coleoptera*	–	PQ423690	PQ569869	PQ569883	PQ569897	Yang et al. 2025a
*O. liaoningensis*	HKAS 132189	Larva of *Coleoptera*	PQ424968	PQ423691	PQ569870	PQ569884	PQ569898	Yang et al. 2025a
*O. liaoningensis*	HKAS 132276	Larva of *Coleoptera*	PQ424969	PQ423692	PQ569871	PQ569885	PQ569899	Yang et al. 2025a
*O. linyphiidarum*	HKAS 132260	* Linyphiidae *	PV139231	PV139247	PV156010	PV155981	–	Yang et al. 2025b
*O. linyphiidarum*	HKAS 132196	* Linyphiidae *	PV139230	PV139246	PV156009	PV155980	PV155995	Yang et al. 2025b
*O. linyphiidarum*	HKAS 132197	* Linyphiidae *	PV139229	PV139245	PV156008	PV155979	PV155994	Yang et al. 2025b
*O. llloydii*	OSC 151913	*Hymenoptera*: *Camponotus*	KJ878924	KJ878891	KJ878970	KJ879004	–	Quandt et al. 2014
*O. longissima*	NBRC 106965	–	AB968392	AB968420	AB968584	–	AB968546	Ban et al. 2015
*O. longissima*	TNC F18448	–	KJ878925	KJ878892	KJ878971	KJ879005	–	Quandt et al. 2014
*O. longistipes*	KUNCC 5224	Termite	OR082949	OR015967	OR030530	OR062224	OR113082	Fan et al. 2024
*O. longistipes*	HKAS 126187	Termite	OR082948	OR015965	OR030529	OR062223	–	Fan et al. 2024
*O. macroacicularis*	BCC 22918	* Lepidoptera *	–	MF614655	MF614639	MF614669	MF614675	Luangsa-ard et al. 2018
*O. macroacicularis*	NBRC 100685	–	AB968388	AB968416	AB968574	–	AB968536	Ban et al. 2015
*O. macroacicularis*	NBRC 105888	* Hepialidae *	AB968389	AB968417	AB968575	–	AB968537	Ban et al. 2015
*O. macroacicularis*	NBRC 105889	* Hepialidae *	AB968390	AB968418	AB968576	–	AB968538	Ban et al. 2015
*O. macroacicularis*	TNS F18550	–	KJ878911	KJ878875	KJ878959	–	–	Quandt et al. 2014
*O. melolonthae*	Ophgrc679	–	–	KC610768	KC610744	KF658666	–	Sanjuan et al. 2015
*O. melolonthae*	OSC 110993	* Coleoptera *	–	–	DQ522331	DQ522376	–	Spatafora et al. 2007
*O. monacidis*	MF74	* Hymenoptera *	KX713647	KX713605	–	KX713712	–	Araújo et al. 2018
*O. mosingtoensis*	BCC 30904	Termite	–	MH753686	MK284273	MK214115	MK214100	Tasanathai et al. 2019
*O. mosingtoensis*	BCC 36921	Termite	–	MH753685	MK284272	MK214116	MK214099	Tasanathai et al. 2019
*O. multiperitheciata*	BCC 22861	* Lepidoptera *	–	MF614656	MF614640	MF614670	MF614683	Luangsa-ard et al. 2018
*O. multiperitheciata*	BCC 69008	* Lepidoptera *	–	MF614657	MF614641	–	MF614682	Luangsa-ard et al. 2018
*O. muscae*	BCC 73616	*Musca domestica*	–	PV257646	PV274278	–	PV274290	Mongkolsamrit et al. 2025
*O. muscae*	BCC 73607	*Musca domestica*	–	PV257645	PV274277	–	PV274289	Mongkolsamrit et al. 2025
*O. muscidarum*	HKAS 132178	*Diptera*: *Muscidae*	PQ424972	PQ423695	PQ675604	–	PQ569900	Yang et al. 2025a
*O. muscidarum*	HKAS 132275	*Diptera*: *Muscidae*	PQ424973	PQ423696	PQ675605	–	PQ569901	Yang et al. 2025a
*O. musicaudata*	SY22072879	* Lepidoptera *	–	OR527545	OR526349	OR526352	–	Peng et al. 2024
*O. myrmecophila*	CEM 1710	–	KJ878928	KJ878894	KJ878974	KJ879008	–	Peng et al. 2024
*O. myrmecophila*	TNS 27120	–	KJ878929	KJ878895	KJ878975	KJ879009	–	Quandt et al. 2014
*O. naomipierceae*	DAWKSAN	* Hymenoptera *	KX713664	KX713589	–	KX713701	–	Araújo et al. 2018
*O. neocommunis*	GZCC 24-0158	Termite	PQ424971	PQ423694	PQ569873	PQ569887	PQ569903	Yang et al. 2025a
*O. neocommunis*	HKAS 13223	Termite	PQ424970	PQ423693	PQ569872	PQ569886	PQ569902	Yang et al. 2025a
*O. neovolkiana*	OSC 151903	–	KJ878930	KJ878896	KJ878976	–	–	Quandt et al. 2014
*O. nigrella*	EFCC 9247	* Lepidoptera *	EF468963	EF468818	EF468758	EF468866	EF468920	Sung et al. 2007
*O. nooreniae*	BRIP 55363	* Hymenoptera *	KX673811	KX673810	KX673812	–	KX673809	Crous et al. 2016
*O. nujiangensis*	YFCC 8880	* Hepialidae *	ON723384	ON723381	ON868820	ON868823	ON868826	Sun et al. 2022
*O. nujiangensis*	YHH 20041	* Lepidoptera *	ON723385	ON723383	ON868822	ON868825	ON868827	Sun et al. 2022
*O. nutans*	NBRC 100944	–	JN941713	JN941428	AB968588	–	AB968549	Ban et al. 2015
*O. nutans*	OSC 110994	Stink bug	DQ522549	DQ518763	DQ522333	DQ522378	–	Spatafora et al. 2007
*O. ootakii*	J13	*Hymenoptera*: *Polyrhachis moesta*	KX713652	KX713600	KX713681	KX713708	–	Araújo et al. 2018
*O. ovatospora*	YHH 2206001	–	–	OP295113	OP313801	OP313803	OP313805	Tang et al. 2022
*O. ovatospora*	YFCC 22069184	–	OP295111	OP295114	OP313802	OP313804	–	Tang et al. 2022
*O. pauciovoperitheciata*	BCC 39781	–	–	MF614650	MF614635	MF614667	MF614671	Luangsa-ard et al. 2018
*O. pauciovoperitheciata*	BCC 45562	–	–	MF614651	MF614634	MF614666	MF614674	Luangsa-ard et al. 2018
*O. ponerus*	XCH ant 03	* Hymenoptera *	KY953152	–	KY953153	KY953154	–	Qu et al. 2018
*O. pruinosa*	NHJ 12994	* Hemiptera *	EU369106	EU369041	EU369024	EU369063	EU369084	Johnson et al. 2009
*O. pseudoacicularis*	BCC 49256	*Hymenoptera*: ant	–	MF614645	MF614629	MF614662	MF614676	Luangsa-ard et al. 2018
*O. pseudoacicularis*	BCC 53843	*Hymenoptera*: ant	–	MF614646	MF614630	MF614661	MF614677	Luangsa-ard et al. 2018
*O. pseudorhizoidea*	BCC 48879	Termite	–	MH753673	MK284261	MK214104	MK214089	Tasanathai et al. 2019
*O. pseudorhizoidea*	BCC 86431	Termite	–	MH753674	MK284262	MK751469	MK214090	Tasanathai et al. 2019
*O. puluongensis*	YFCC 6442	Termite	MT141118	MT270528	MT270520	MT270523	MT270526	Xu et al. 2022
*O. puluongensis*	YFCC 6443	Termite	MT141119	MT270529	MT270521	MT270524	MT270527	Xu et al. 2022
*O. puluongensis*	YHH 16017	Termite	–	MT270530	MT270522	MT270525	–	Xu et al. 2022
*O. pulvinata*	TNS F 30044	*Hymenoptera*: ant	GU904208	AB721305	GU904209	GU904210	–	Kepler et al. 2011
*O. purpureostromata*	TNS F18430	* Coleoptera *	KJ878931	KJ878897	KJ878977	KJ879011	–	Quandt et al. 2014
*O. radiata*	HKAS 135613	* Diptera *	–	PP958850	PP956622	–	–	Xie et al. 2025
*O. radiciformis*	BCC 93036	Termite	–	MZ675425	MZ707852	MZ707857	MZ707808	Tasanathai et al. 2022
*O. ramosissimum*	GZUH2012HN2	*Hepialidae*: *Endoclita*	KJ028013	–	KJ028016	KJ028018	–	Wen et al. 2014
*O. ramosissimum*	GZUHHN8	*Phassus nodus*	KJ028012	–	KJ028014	KJ028017	–	Wen et al. 2014
*O. ravenelii*	OSC 151914	–	KJ878932	–	KJ878978	KJ879012	KJ878950	Quandt et al. 2014
*O. rubiginosiperitheciata*	NBRC 100946	–	JN941705	JN941436	AB968581	JN992439	AB968543	Schoch et al. 2012
*O. rubiginosiperitheciata*	NBRC 106966	–	JN941704	JN941437	AB968582	JN992438	AB968544	Schoch et al. 2012
*O. salganeicola*	JPMA107	–	MT741703	MT741716	MT759574	MT759577	–	Araujo et al. 2020
*O. salganeicola*	Mori01	–	MT741705	MT741719	MT759575	MT759578	MT759580	Araujo et al. 2020
*O. satoi*	J19	*Polyrhachis lamellidens*	KX713650	KX713601	KX713684	KX713710	–	Araújo et al. 2018
***O. simaoensis***	**HKAS 126113**	***Gryllotalpa (Orthoptera)***	**PX852565**	**PX852568**	**PX893080**	**PX893075**	**PX893072**	**This study**
***O. simaoensis***	**HKAS 126114**	***Gryllotalpa (Orthoptera)***	**–**	**PX852569**	**PX893081**	**PX893076**	**–**	**This study**
***O. simaoensis***	**KUNCC 11568**	***Gryllotalpa (Orthoptera)***	**PX852566**	**–**	**PX893082**	**PX893077**	**–**	**This study**
*O. sinensis*	QH06-197	* Hepialidae *	JX968025	JX968030	JX968015	JX968005	JX968010	Zhang et al. 2013
*O. sinensis*	QH09-201	* Hepialidae *	JX968024	JX968029	JX968014	JX968004	JX968009	Zhang et al. 2013
*O. sinensis*	XZ06-44	* Hepialidae *	JX968026	JX968031	JX968016	JX968006	JX968011	Zhang et al. 2013
*O. sinensis*	YN07-8	* Hepialidae *	JX968027	JX968032	JX968017	JX968007	JX968012	Zhang et al. 2013
*O. sinensis*	YN09-64	* Hepialidae *	JX968028	JX968033	JX968018	JX968008	JX968013	Zhang et al. 2013
*O. sinensis*	CUHK CSC2	* Hepialidae *	–	HM595902	HM595936	HM595968	–	Chan et al. 2011
*O. sinocampes*	GZUIFR 2010MC-1	* Lepidoptera *	–	PQ766190	PQ787212	–	PQ787213	Xu et al. 2025
*O. sinocampes*	GZUIFR2022MLH-H1	* Coccoidea *	PQ497592	PQ497632	PQ516628	PQ516630	PQ516634	Xu et al. 2025
*O. sinocampes*	GZUIFR-2022MLHH1C	* Coccoidea *	PQ497593	PQ497633	PQ516629	PQ516631	PQ516635	Xu et al. 2025
*O. sobolifera*	KEW 78842	* Cicadidae *	EF468972	EF468828	–	EF468875	EF468925	Sung et al. 2007
*O. sobolifera*	NBRC 106967	* Cicadidae *	AB968395	AB968422	AB968590	–	AB968551	Ban et al. 2015
*O. spataforae*	MY11765	–	–	MG831747	MG831746	MG831748	MG831749	Luangsa-ard et al. 2018
*O. spataforae*	OSC 128575	* Hemiptera *	EF469126	EF469079	EF469064	EF469093	EF469110	Sung et al. 2007
*O. spataforae*	NHJ 12525	* Fulgoridae *	–	EF469078	EF469063	EF469092	EF469111	Sung et al. 2007
*O. sphecocephala*	NBRC 101416	–	JN941698	JN941443	–	JN992432	–	Schoch et al. 2012
*O. spicatus*	MFLU 18-0164	*Coleoptera*: *Tenebrionoidea*	MK863047	MK863054	MK860192	–	–	Zha et al. 2021
*O. stylophora*	OSC 111000	*Coleoptera*: *Elateridae*	DQ522552	DQ518766	DQ522337	DQ522382	DQ522433	Spatafora et al. 2007
*O. stylophora*	NBRC 100947	–	JN941694	JN941447	AB968579	JN992428	AB968541	Schoch et al. 2012
*O. stylophora*	NBRC 100948	–	JN941693	JN941448	AB968580	JN992427	AB968542	Schoch et al. 2012
*O. stylophora*	NBRC 100949	–	JN941692	JN941449	–	JN992426	–	Schoch et al. 2012
*O. stylophora*	OSC 110999	–	EF468982	EF468837	EF468777	EF468882	EF468931	Sung et al. 2007
*O. stylophora*	OSC 111000	* Coleoptera *	DQ522552	DQ518766	DQ522337	DQ522382	DQ522433	Spatafora et al. 2007
*O. tabani*	BCC 45127	* Tabanus *	–	PV257652	–	–	PV339938	Mongkolsamrit et al. 2025
*O. tabani*	BCC 39918	* Tabanus *	–	PV257651	PV274284	–	–	Mongkolsamrit et al. 2025
*O. termiticola*	BCC 1920	Termite	–	MH753678	MK284265	MK214108	MK214094	Tasanathai et al. 2019
*O. termiticola*	BCC 1770	Termite	–	MH753677	MK284264	MK214107	MK214093	Tasanathai et al. 2019
*O. thanathonensis*	MFU 16-2909	–	–	MF850378	MF872613	MF872615	–	Xiao et al. 2017
*O. thilosuensis*	BCC 47494	*Anastrepha obliqua*	–	PV257653	PV274285	–	PV274294	Mongkolsamrit et al. 2025
*O. thilosuensis*	BCC 46607	*Anastrepha obliqua*	–	PV257654	PV274286	–	–	Mongkolsamrit et al. 2025
*O. tiputini*	Ophsp. 11465	–	KC610792	KC610773	KC610745	KF658671	–	Sanjuan et al. 2015
*O. tricentri*	NBRC 106968	–	AB968393	AB968423	AB968593	–	AB968554	Ban et al. 2015
*O. tielingensis*	HKAS 135612^T^	* Lepidoptera *	PP951446	PP958848	PP956621	PP966945	PP955355	Xie et al. 2025
*O. unilateralis*	Ophuni866	–	KC610799	–	KC610742	KF658674	KC610718	Sanjuan et al. 2015
*O. unilateralis*	OSC 128574	* Hymenoptera *	DQ522554	DQ518768	DQ522339	DQ522385	DQ522436	Spatafora et al. 2007
*O. unilateralis*	SERI1	*Camponotus sericeiventris*	KX713628	KX713626	KX713675	KX713730	–	Araújo et al. 2018
*O. unituberculata*	YFCC HU1301	*Lepidoptera*: *Noctuidae*	KY923213	KY923211	KY923215	KY923217	–	Wang et al. 2018
*O. unituberculata*	YHH HU1301	*Lepidoptera*: *Noctuidae*	KY923214	KY923212	KY923216	KY923218	–	Wang et al. 2018
*O. variabilis*	ARSEF 5365	* Diptera *	DQ522555	DQ518769	DQ522340	DQ522386	DQ522437	Spatafora et al. 2007
*O. xuefengensis*	GZUH2012HN13	*Phassus nodus*	KC631787	–	KC631792	KC631797	–	Wen et al. 2013
*O. xuefengensis*	GZUH2012HN14	*Phassus nodus*	KC631789	–	KC631793	KC631798	–	Wen et al. 2013
*O. yakusimensis*	HMAS 199604	* Cicadidae *	KJ878938	KJ878902	–	KJ879018	KJ878953	Quandt et al. 2014
*Ophiocordyceps* sp.	TNS 16250	* Coleoptera *	KJ878942	–	KJ878987	KJ879021	–	Quandt et al. 2014
*Ophiocordyceps* sp.	TNS 16252	–	KJ878941	KJ878906	KJ878986	–	–	Quandt et al. 2014
*Ophiocordyceps* sp.	NHJ 12581	* Lepidoptera *	EF468973	EF468831	EF468775	–	EF468930	Sung et al. 2007
*Ophiocordyceps* sp.	NHJ 12582	* Lepidoptera *	EF468975	EF468830	EF468771	–	EF468926	Sung et al. 2007
*Ophiocordyceps* sp.	OSC 110997	–	EF468976	–	EF468774	EF468879	EF468929	Sung et al. 2007
*Paraisaria amazonica*	Ophama2026	–	KJ917562	KJ917571	KM411989	KP212902	KM411982	Sanjuan et al. 2015
*Par. blattarioides*	HUA 186093	* Blattodea *	KJ917559	KJ917570	KM411992	KP212910	–	Sanjuan et al. 2015
*P. coenomyiae*	NBRC 108993	–	AB968384	AB968412	AB968570	–	AB968532	Ban et al. 2015
*P. gracilioides*	Ophgrc934	–	KJ917556	–	–	KP212914	–	Sanjuan et al. 2015
*P. gracilis*	EFCC 3101	* Lepidoptera *	EF468955	EF468810	EF468750	EF468858	EF468913	Sung et al. 2007
*P. gracilis*	EFCC 8572	* Lepidoptera *	EF468956	EF468811	EF468751	EF468859	EF468912	Sung et al. 2007
*P. heteropoda*	NBRC 100642	–	JN941720	JN941421	AB968594	–	AB968555	Ban et al. 2015
*P. orthopterorum*	BBC 88305	*Orthoptera* (nymph)	–	MK332583	MK214080	MK214084	–	Mongkolsamrit et al. 2019
*P. phuwiangensis*	TBRC 9709	*Coleoptera*: *Elateridae*	–	MK192057	MK214082	MK214086	–	Mongkolsamrit et al. 2019
*P. tettigonia*	GZUHCS14062709	* Tettigoniidae *	KT345955	–	KT375440	KT375441	–	Wen et al. 2014
*P. yodhathai*	BBH 43163	*Coleoptera*: *Elateridae*	–	MK332584	MH211353	MH211349	–	Mongkolsamrit et al. 2019

Note: New species were shown in bold.

## Results

### Sequencing and phylogenetic analyses

**Figure 1.**
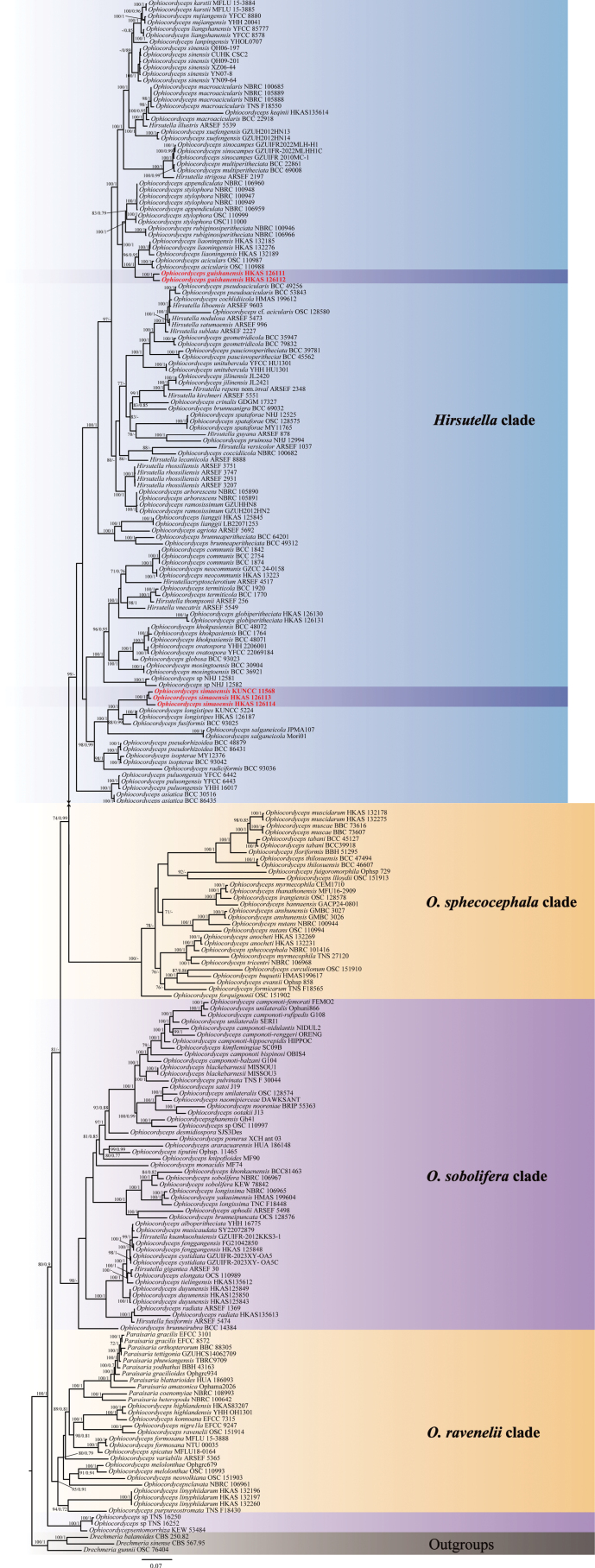
Phylogenetic tree inferred from a multi-gene (nr*SSU*, nr*LSU*, *tef-1α*, *rpb1* and *rpb2*) dataset of 239 taxa using ML and BI methods. Statistical support values of greater than 70% are provided at the nodes for ML bootstrap proportions and BI posterior probabilities. Newly described taxa in this study are highlighted in bold.

The combined dataset comprising 239 samples was used for the ML and the BI phylogenetic analyses to determine the phylogenetic placement of the new species in *Ophiocordyceps*. Three species of *Drechmeria* W. Gams & H.-B. Jansson in *Clavicipitaceae*, namely *D. balanoides* (Drechsler) Spatafora & Kepler, *D. gunnii* (Berk.) Spatafora, Kepler & C.A. Quandt, and *D. sinensis* (K.Q. Zhang, L. Cao & Z.Q. Liang) Spatafora & Kepler, were designated as the outgroup taxa. The concatenated five-gene dataset contained 4,810 bp (1,088 bp for nr*SSU*, 922 bp for nr*LSU*, 998 bp for *tef-1α*, 768 bp for *rpb1*, and 1,034 bp for *rpb2*). Phylogenetic trees constructed using ML and BI methods showed consistent topologies with most well-supported branches (Fig. 1). Samples HKAS 126113, HKAS 126114, and KUNCC 11568, newly described here as *O. simaoensis*, formed a distinct and well-supported clade (BP = 100%, PP = 1) and were closely related to *O. longistipes* Y.B. Wang et al. and *O. fusiformis* Tasan. et al. Meanwhile, samples HKAS 126111 and HKAS 126112, proposed as *O. guishanensis*, formed a well-supported monophyletic clade within *Ophiocordyceps* (BP = 100%, PP = 1), closely related to *O. acicularis* (Ravenel) Petch and *O. liaoningensis* Y.P. Xiao et al.

## Taxonomy

### Ophiocordyceps simaoensis

Taxon classificationFungiOphiocordycipitaceae

Z.Q. Wang & Y.B. Wang sp. nov.

CB673D0E-6482-5EFC-A7A7-051BA9B0A560

861273

Fig. 2

#### Etymology.

Referring to the location where the fungus was discovered.

**Figure 2.**
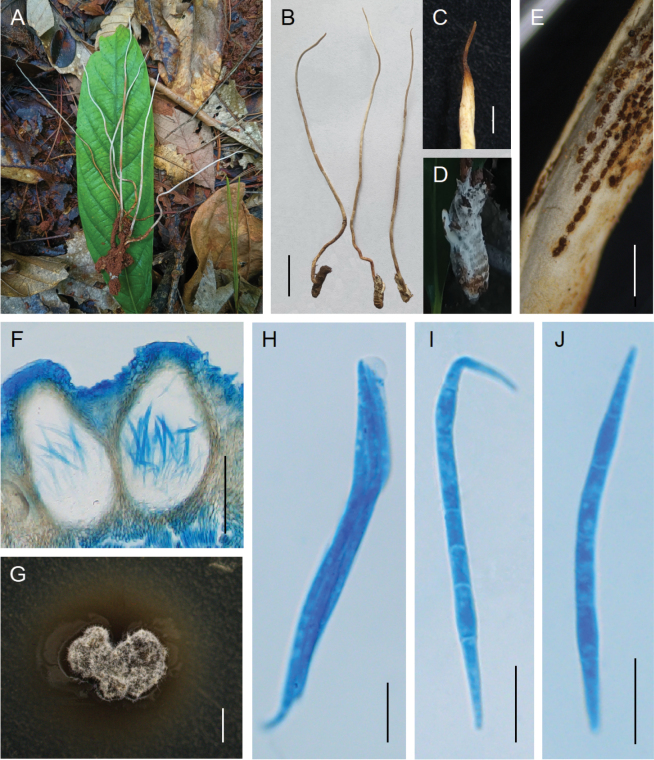
*Ophiocordyceps simaoensis*. **A, B**. Stromata of fungus arising from the hosts; **C**. Apex of stroma; **D**. The host *Gryllotalpa* sp.; **E, F**. Perithecia; **G**. Colony on PDA; **H**. Asci; **I, J**. Ascospores. Scale bars: 2 cm (**B**); 1 mm (**C**); 500 µm (**E**); 100 µm (**F**); 1 mm (**G**); 10 µm (**H–J**).

#### Type.

China • Yunnan Province, Pu’er City, Simao District, Ancient Tea Horse Road. 22°50'N, 100°59'E, alt. 1534 m, on an adult of *Gryllotalpa* buried in soil. 03 Aug. 2025, Zhiqin Wang (holotype HKAS 126113, ex-type culture KUNCC 11568).

#### Diagnosis.

Similar to *Ophiocordyceps fusiformis*, but differs from it by producing smaller perithecia, asci, and ascospores. Its fertile parts are intermittent and irregular sheets distributed in the middle part of the stromata.

#### Description.

Stromata arising from dorsum or lateral side of insects buried in soil at a depth of about 8 cm. Stromata solitary, unbranched, cylindrical, flexible, leathery, 13–19 cm long, 0.8–1.5 mm wide, greyish white to yellowish brown, tapering abruptly toward the apex, with a blackish brown apex of 0.1–0.6 cm long. Fertile parts cylindrical, yellowish brown, 4.4–8.9 cm long, with intermittent and irregular sheets on the middle part of stromata, with a sterile apex of 3.2–4.6 cm. Perithecia pseudoimmersed, dense, vertically arranged, oval to oblong, 209–238 × 105–143 µm. Tip ostioles slightly protruding, yellowish brown at an early stage and blackish brown at maturity. Asci 8-spored, filiform, hyaline, 61–103 × 2.9–7.4 µm, with a hemispheric apical cap of 3.6–5.1 × 1.9–2.6 µm. Ascospores filiform, multiseptate, hyaline, tapering at both ends, 42–52 × 2.2–2.9 µm, not breaking into secondary ascospores. **Asexual morph**: Undetermined.

#### Cultural characteristics.

Colonies on PDA grow slowly, reaching 3 mm in diameter about 50 days at 25 °C, dark brown, with a layer of white mycelia covering the surface.

#### Habitat.

Parasitic on adults of *Gryllotalpa* (*Gryllotalpidae*, *Orthoptera*) buried in soil of the evergreen broad-leaved forests.

#### Distribution.

China, Yunnan Province, Pu’er City.

#### Other material examined.

China • Yunnan Province, Pu’er City, Simao District, Ancient Tea Horse Road. 22°50'N, 100°59'E, alt. 1531 m, on an adult of *Gryllotalpa* buried in soil. 03 Aug. 2025, Zhiqin Wang (HKAS 126114, paratype; KUNCC 11569, ex-paratype culture).

#### Commentary.

Phylogenetic analyses showed that the three samples of *O. simaoensis* were clustered as a distinct clade with a close relationship to *O. longistipes* and *O. fusiformis* (Fig. 1). However, these species differ in their host associations: *O. simaoensis* is parasitic on adults of *Gryllotalpa*, whereas *O. longistipes* and *O. fusiformis* are parasitic on termites. Morphologically, a key distinguishing feature is the position of perithecia: pseudoimmersed in *O. simaoensis* versus superficial in *O. longistipes*. Additionally, *O. simaoensis* has smaller perithecia (209–238 × 105–143 µm vs. 390–420 × 295–350 µm), shorter asci (61–103 × 2.9–7.4 µm vs. 160–195 × 4.5–6.5 µm), and shorter ascospores (42–52 × 2.2–2.9 µm vs. 70–85 × 3.5–4.5 µm) than *O. longistipes* (Fan et al. 2024). Similarly, *O. simaoensis* is distinguished from *O. fusiformis* by its pseudoimmersed perithecia, smaller perithecia, shorter asci and ascospores (Table 2).

**Table 2.** Morphological comparison between new species and related species.

Species	Stromata (mm)	Fertile parts (mm)	Perithecia (µm)	Asci (μm)	Ascospores (μm)	Phialides (μm)	Conidia (μm)	References
*Ophiocordyceps acicularis*	—	—	Black, broadly conical or ovate, 280 × 250	Narrow-clavate, 260–290 × 7–10	Linear-clavate, 150–240 × 3–4	—	—	Petch 1933
*O. fusiformis*	Solitary, cylindrical, brown, 60 × 1–2	14–15 × 4–5	Superficial, ovoid, 316.5–347.5 × 186.5–232	8-spored, cylindrical, 170.5–210.5 × 8–11	Cylindrical, 49–67 × 5–6	Monophialidic, 11–21 × 2–4	Fusiform, 7–13.5 × 2–4	Tasanathai et al. 2022
***O. guishanensis***	**Solitary, unbranched, cylindrical, yellowish-brown to black-brown, 56–87 × 0.3–0.5**	**Cylindrical, yellowish-brown, 19–35 long**	**Superficial, black-brown to black, oval to oblong, 250–584 × 173–542**	**8-spored, filiform, 86–145 × 2.1–6.1**	**Not observed**.	**—**	**—**	**This study**
*O. liaoningensis*	Single, cylindrical, pale brown, 50–60 × 1–3	Cylindrical, dark brown, 10–20 × 2–3	Superficial, dark brown, ovoid to flask-shaped, 310–415 × 170–290	8-spored, cylindrical, 205–255 × 7–11	Slender filiform, 150–200 × 2–4	Polyblastic, clavate or bottle-shaped, 15–33 × 3.5–6.5	Ovoid or subglobose, 5–9 × 4.2–6.4	Yang et al. 2025
*O. longistipes*	Solitary, unbranched, cylindrical, grayish white to yellowish brown, 170–240 × 0.5–1.0	Cylindrical, yellowish brown, 30–55 long	Superficial, pale yellow to brown, pyramidal to oval, 390–420 × 295–350	8-spored, filiform, 160–195 × 4.5–6.5	Filiform, 70–85 × 3.5–4.5	Monophialidic or rarely polyphialidic, flaskshaped, 29–60 × 4–4.5	Citriform or oval, 7–10 × 4.5–7	Fan et al. 2024
***O. simaoensis***	**Solitary, unbranched, cylindrical, greyish white to yellowish brown, 130–190 × 0.8–1.5**	**Cylindrical, yellowish brown, 44–89 long**	**Pseudo-immersed, yellowish brown to blackish brown, oval to oblong, 209–238 × 105–143**	**8-spored, filiform, 61–103 × 2.9–7.4**	**Filiform, 42–52 × 2.2–2.9**	**—**	**—**	**This study**

The symbol “—” means that the data is unavailable.

### Ophiocordyceps guishanensis

Taxon classificationFungiOphiocordycipitaceae

Z.Q. Wang & Y.B. Wang sp. nov.

7F0124A1-E82C-5D63-A337-6D9A258B37B3

861274

Fig. 3

#### Etymology.

Named after the name Guishan National Forest Park, where the species was discovered.

**Figure 3.**
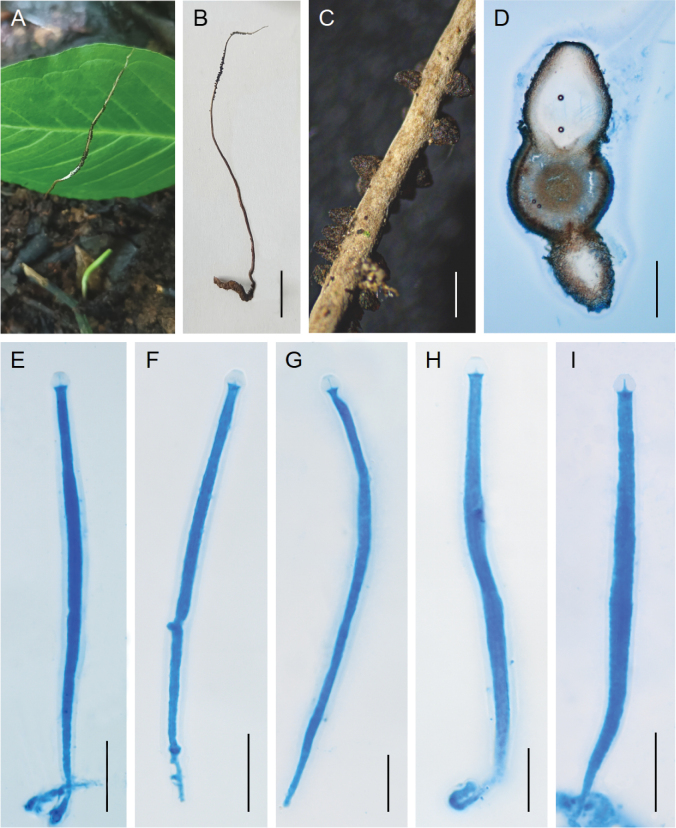
*Ophiocordyceps guishanensis*. **A, B**. Stromata of fungus arising from the hosts; **C**. Fertile part; **D**. Perithecia; **E–I**. Asci. Scale bars: 1 cm (**B**); 500 µm (**C**); 200 µm (**D**); 20 µm (**E–I**).

#### Type.

China • Yunnan Province, Kunming City, Shilin County, the Guishan National Forest Park. 24°64'N, 103°60'E, alt. 2284 m, on a larva of *Elateridae* buried in soil. 19 Jul. 2025, Zhiqin Wang (HKAS 126111, holotype).

#### Diagnosis.

Similar to *Ophiocordyceps acicularis*, but differs from it by producing smaller asci.

#### Description.

**Sexual morph**: Stromata arising from the head of coleopteren larva buried in soil, solitary, unbranched, cylindrical, flexible, leathery, 5.6–8.7 cm long, 0.3–0.5 mm wide, yellowish-brown to black-brown, colouration from a darker base to a lighter apex, tapering gradually toward the apex. Fertile parts cylindrical, yellowish-brown, 1.9–3.5 cm long, distributed intermittently and irregularly over the upper portion of the stromata, with a sterile apex of 3.5–5.1 mm. Perithecia superficial, black-brown to black, loosely aggregated and arranged in a disordered, vertical orientation, oval to oblong, 250–584 × 173–542 µm. Asci 8-spored, filiform, hyaline, 86–145 × 2.1–6.1 µm, with a hemispheric apical cap of 1.6–6.1 × 1.8–5.9 µm. Ascospores not observed. **Asexual morph**: Undetermined.

#### Habitat.

Parasitic on larvae of *Elateridae (Coleoptera)* buried in soil.

#### Distribution.

China, Yunnan Province, Kunming City, Shilin County, the Guishan National Forest Park.

#### Other material examined.

China • Yunnan Province, Shilin County, the Guishan National Forest Park. 24°64'N, 103°60'E, alt. 2284 m, on a larva of *Elateridae* buried in soil. 19 Jul. 2025, Zhiqin Wang (HKAS 126112, paratype).

#### Commentary.

Phylogenetic analyses revealed that two samples of *O. guishanensis* were grouped together and formed a distinct clade within the *Hirsutella* clade of *Ophiocordyceps*, showing close relationship to *O. acicularis* and *O. liaoningensis* (Fig. 1). All three species are parasites of the larvae of *Elateridae*. Morphologically, *Ophiocordyceps guishanensis* produces smaller asci (86–145 × 2.1–6.1 µm) compared to *O. acicularis* (260–290 × 7–10 µm) and *O. liaoningensis* (205–255 × 7–11 µm) (Table 2).

## Discussion

*Ophiocordyceps* species are globally distributed across diverse forest ecosystems, particularly in tropical and subtropical regions (Xu et al. 2025). With a broad host range, they have been reported to infect various insects, including *Blattaria*, *Coleoptera*, *Dermaptera*, *Diptera*, *Hemiptera*, *Hymenoptera*, *Isoptera*, *Lepidoptera*, *Megaloptera*, *Mantodea*, *Odonata*, and *Orthoptera* (Yang et al. 2025a). In this study, *O. guishanensis* and *O. simaoensis* were described; they were parasitizing insects of *Elateridae (Coleoptera)* and *Gryllotalpa* (*Gryllotalpidae*, *Orthoptera*), respectively. *Coleoptera* is one of the most species-rich insect orders comprising 176 families and an estimated 350,000 to 400,000 described species worldwide (Bouchard et al. 2017). Among these, eleven families, namely *Scarabaeidae*, *Geotrupidae*, *Lucanidae*, *Elateridae*, *Cerambycidae*, *Chrysomelidae*, *Erotylidae*, *Curculionidae*, *Tenebrionidae*, *Staphylinidae*, and *Carabidae*, are known hosts of *Cordyceps*-like fungi (Shrestha et al. 2016).

Thirty species of *Ophiocordyceps* were documented as parasites of *Coleoptera* in the previous review about hosts of *Cordyceps* s.l. (Shrestha et al. 2016). Among these, five species are parasitic on *Elateridae*: *O. brunneipunctata* (Hywel-Jones) G.H. Sung et al., *O. elateridicola* (Kobayasi & Shimizu) G.H. Sung et al., *O. purpureostromata* (Kobayasi) G.H. Sung et al., *O. salebrosa* (Mains) G.H. Sung et al., and *O. stylophora* (Berk. & Broome) G.H. Sung et al. Subsequent studies have expanded this number, currently about 60 species of *Ophiocordyceps* are parasitic on *Coleoptera* (Mongkolsamrit et al. 2024). To date, more than ten species have been found as parasites of *Elateridae (Coleoptera)*. These species are sporadically distributed in the phylogenetic tree of *Ophiocordyceps* and do not form a monophyletic group (Lao et al. 2021; Wang et al. 2021b; Zou et al. 2022; Mongkolsamrit et al. 2024; Yang et al. 2025b).

Our phylogenetic analyses indicate that *Ophiocordyceps* comprises four clades, namely the *Hirsutella*, *O. sobolifera*, *O. sphecocephala*, and *O. ravenelii* clades. Most species parasitizing *Elateridae (Coleoptera)* are concentrated in the *O. sobolifera* and *Hirsutella* clades. Two species on *Elateridae*, *O. borealis* and *O. purpureostromata*, belong to the *O. ravenelii* clade. Within the *Hirsutella* clade, *O. acicularis*, *O. liaoningensis*, *O. stylophora*, and *O. guishanensis* on *Elateridae* group together and form a distinct clade. Morphologically, species in the *O. sobolifera* and *O. ravenelii* clades generally possess immersed or completely immersed perithecia, with the exception of *O. sporangifera*, in which only synnemata were observed. Within the *Hirsutella* clade, species associated with *Elateridae (Coleoptera)* share similar morphological characteristics: solitary cylindrical stromata with a sterile apex and superficial perithecia.

Four *Ophiocordyceps* species have been reported to parasitize *Gryllotalpa* spp., namely *O. gryllotalpae* Petch, *O. krachonicola* Tasan., Thanakitp. & Luangsa-ard, *O. neogryllotalpae* Y.P. Xiao & Yu Yang, and *O. monticola* (Mains) G.H. Sung et al. (Mains 1940; Kobayasi 1941; Thanakitpipattana et al. 2020; Yang et al. 2024). Morphologically, *O. krachonicola* and *O. monticola* are characterized by completely immersed perithecia and fractured ascospores. *Ophiocordyceps gryllotalpae* has superficial perithecia and exhibits shorter stromata than *O. simaoensis*. Perithecia of *O. simaoensis* and *O. neogryllotalpae* are pseudoimmersed and semi-immersed, respectively. Furthermore, a prominent distinguishing feature is that the stromata of *O. simaoensis* have blackish-brown apices and fertile parts with intermittent and irregular sheets on the middle part, a characteristic absent in *O. neogryllotalpae*.

The fungal hosts, *Gryllotalpa* spp., commonly known as “mole crickets”, inhabits soil environments and exhibits a broad dietary range. As notorious pests in agriculture, forestry, and horticulture, they damage crops by consuming plant roots, stems, and leaves. Our specimen collection reveals high infection rates in field populations of *Gryllotalpa*, suggesting this fungus has considerable potential for population control. Therefore, future investigations into entomopathogenic fungi parasitizing *Gryllotalpa* species are recommended to conserve biological resources and support the development of sustainable, eco-friendly biological control strategies.

## Supplementary Material

XML Treatment for Ophiocordyceps simaoensis

XML Treatment for Ophiocordyceps guishanensis

## Additional information

### Use of AI

No use of AI was reported.

### Author ORCIDs

Zhi-Qin Wang https://orcid.org/0000-0001-9022-3635

Yuan-Bing Wang https://orcid.org/0000-0002-3305-9418

### Data availability

All of the data that support the findings of this study are available in the main text.
